# Change in Antithyroglobulin Antibody Levels is a Good Predictor of Responses to Therapy in Antithyroglobulin Antibody-Positive Pediatric Papillary Thyroid Carcinoma Patients

**DOI:** 10.1155/2022/7173919

**Published:** 2022-03-10

**Authors:** Chuang Xi, Guo-Qiang Zhang, Hong-Jun Song, Chen-Tian Shen, Li-Ying Hou, Zhong-Ling Qiu, Quan-Yong Luo

**Affiliations:** Department of Nuclear Medicine, Shanghai Jiao Tong University Affiliated Sixth People's Hospital, 600 Yishan Road, Shanghai 200233, China

## Abstract

**Objective:**

Antithyroglobulin antibodies (TgAbs) could be used as a surrogate tumor marker of TgAb-positive-differentiated thyroid carcinoma. This study aims to determine whether the change in TgAb levels over time could be used as a predictor of responses to therapy in pediatric papillary thyroid carcinoma (PTC) patients.

**Methods:**

We retrospectively analyzed the records of 48 pediatric PTC patients with TgAb levels ≥50 IU/ml 6 months after initial ^131^I treatment. Suppressed thyroglobulin (Tg) levels 6 months after initial ^131^I treatment were used to divide the patients into positive Tg (P-Tg, Tg ≥ 0.2 ng/ml) and negative Tg (N-Tg, Tg < 0.2 ng/ml) groups. Responses to therapy were classified as the acceptable response (AR) group and the not acceptable response (NAR) group.

**Results:**

Of 48 enrolled patients with 58 months (range, 24–143 months) of follow-up, 28 patients had NAR and 20 patients had AR. TgAb levels were decreasing ≥50% in 28 patients, decreasing <50% in 8 patients, and increasing in 12 patients. Multivariate analysis showed that high initial risk stratification and TgAb levels decreasing <50% or increasing were significantly associated with NAR (*p* < 0.05). Changes in Tg levels were also associated with NAR in the P-Tg group (*p* < 0.05).

**Conclusion:**

Changes in TgAb levels over time could be used as a predictor of responses to therapy in TgAb-positive pediatric PTC patients. Changes in Tg levels over time are also associated with NAR to therapy in both TgAb-positive and Tg-positive pediatric PTC patients.

## 1. Introduction

Papillary thyroid carcinoma (PTC) is the most common thyroid cancer in both children and adults, accounting for 85%–90% of differentiated thyroid carcinoma (DTC) [1]. Although most PTC patients have an indolent clinical course and a favorable prognosis, 15%–30% of these patients experience recurrence. Thus, a lengthy surveillance period is needed, owing to the risk of recurrence after initial treatment, especially in pediatric PTC patients [2, 3].

Thyroglobulin (Tg) is the main serum marker for DTC, especially when measured during hypothyroidism or after stimulation with recombinant thyroid-stimulating hormone [3]. Its high specificity is based on the fact that normal thyroid tissue or thyroid cancer cells are the only bodily source of Tg [4]. The persistence of or increase in Tg levels after total thyroidectomy and radioactive iodine (^131^I) treatment is a reliable indicator of persistent or recurrent disease in PTC. Antithyroglobulin antibodies (TgAbs) may be produced in response to the secretion of Tg by thyroid tissue or thyroid cancer cells. The prevalence of TgAbs is 15%–25% in patients with DTC, which is approximately twice the level in those without DTC [5, 6]. The presence of TgAbs, even at very low levels, may cause unreliable Tg measurements that can result in an inaccurate diagnosis of persistent or recurrent disease [6, 7]. Therefore, current guidelines recommend that measurement of Tg levels should always be accompanied by a TgAb test in DTC patients [3, 8]. Most studies reported that the de novo appearance, persistence, or increase of TgAbs is a biochemical sign of persistent or recurrent disease in DTC [6, 8–18]. Thus, it is possible to monitor changes in TgAb levels over time as a surrogate tumor marker for DTC.

The prevalence of TgAbs in pediatric DTC patients was higher than in adult DTC patients [6, 7, 19]. Although the prognostic significance of TgAb levels is well demonstrated in adult DTC, its prognostic value in pediatric PTC is still unclear [19]. Pediatric PTC exhibits differences in pathophysiology, clinical presentation, and long-term outcomes compared with adult PTC [2, 20–22]. Thus, it is inappropriate to extrapolate the prognostic value of TgAbs from adult data to pediatric patients. However, to our knowledge, no studies to date have focused on whether increasing TgAb levels could be used as a surrogate tumor marker to predict prognosis in pediatric PTC patients.

Because of the high prevalence of TgAbs in pediatric PTC patients and minimal data reported, we retrospectively analyzed data from TgAb-positive pediatric PTC patients and aimed to determine whether increasing TgAb levels could be used as a predictor of responses to therapy in pediatric PTC patients and describe the clearance pattern of TgAbs in pediatric PTC patients.

## 2. Materials and Methods

### 2.1. Patients and Groups

The study was approved by the ethical committee of Shanghai Sixth's People's Hospital. We retrospectively analyzed the medical records of pediatric patients with PTC (*n* = 181) who underwent total or subtotal thyroidectomy followed by ^131^I treatment from January 2008 to December 2018 in the Department of Nuclear Medicine, Shanghai Jiao Tong University Affiliated Sixth People's Hospital, Shanghai, China. Inclusion criteria were as follows: (i) PTC, (ii) treatment with ^131^I, (iii) age ≤18 years [2], and (iv) positive TgAb status with serum TgAb ≥ 50 IU/ml 6 months after ^131^I treatment [16, 23]. Of the 48 patients finally included in this study, 17 patients with positive Tg (suppressed Tg ≥ 0.2 ng/ml) 6 months after ^131^I treatment were included in the positive Tg (P-Tg) group and 31 patients with negative Tg (suppressed Tg < 0.2 ng/ml) 6 months after ^131^I treatment were included in the negative Tg (N-Tg) group. The median duration of follow-up for the overall study cohort was 58 months (range, 24–143 months).

### 2.2. Procedures for ^131^I Treatment

Before ^131^I treatment, each patient was given a low-iodine diet and began levothyroxine withdrawal for 2–4 weeks to achieve a thyroid-stimulating hormone (TSH) level of＞30 mIU/L. Routine measurement of thyroid-stimulating hormone, Tg, and TgAb; neck ultrasonography; and chest CT scans were performed before oral administration of ^131^I. An empiric ^131^I dosing strategy was used to determine the ^131^I regimen calculated by experts with experience in dosing children [2, 24]. Specifically, an oral dose of 1.11–3.7 GBq (30–100 mCi) ^131^I was administered to patients with unknown distant metastases before initial treatment and an oral dose of 3.7–7.40 GBq (100–200 mCi) ^131^I was administered to patients known to have distant metastases before treatment. At 4 days after ^131^I administration, a ^131^I-whole-body scan was performed. In patients with ^131^I-avid metastases on ^131^I-WBS, repeated treatments with 3.7–7.40 GBq (100–200 mCi) ^131^I were given 4–12 months later. During follow-up, TSH, serum Tg, TgAb, and neck ultrasonography were measured every 6 months, and another imaging test was performed if needed.

### 2.3. Measurement of Tg and TgAb

Serum Tg and TgAb measurements were performed before ^131^I treatment during thyroid hormone withdrawal and every 6 months after ^131^I treatment during follow-up. The same assay was used for the measurement of TgAb throughout the study period. The cobas® analyzer (Roche Diagnostics GmbH, Basel, Switzerland) was used to determine the Tg and TgAb levels using the same high-sensitivity electrochemiluminescence immunoassay method in the same laboratory of our hospital. The analytical limit of Tg was 0.04 *μ*g/mL with a detection range of 0.04 to 25,000 ng/mL, and the analytical limit of TgAb was 10 IU/ml with a detection range of 10 to 4,000 IU/ml.

### 2.4. Dynamic Changes in TgAb and Tg Levels

The changes in TgAb levels were calculated by ([TgAb level at the end of follow-up] − [TgAb level 6 months after initial ^131^I treatment])∕(TgAb level 6 months after initial ^131^I treatment). The changes in TgAb levels were defined in accordance with previous studies as follows [18, 25–27]:

- Increasing: increase in TgAb levels.
- Decreasing <50%: decrease in TgAb levels by less than 50%.
- Decreasing ≥50%: decrease in TgAb levels by more than 50%.

Besides, TgAb clearance was defined as TgAb decreased to <50 IU/ml during follow-up [16, 23]. The definition of the changes in Tg was similar to TgAb.

### 2.5. Responses to Therapy Assessments

The responses to therapy were assessed on the basis of serum Tg and TgAb measurements and imaging tests [3, 17]. The responses to therapy were defined as follows:

- Excellent response (ER): suppressed Tg < 0.2 ng/ml or stimulated Tg < 1 ng/ml, TgAb < 50 IU/ml, and negative imaging.
- Indeterminate response (IR): suppressed Tg between 0.2 and 1 ng/mL or stimulated Tg between 1 and 10 ng/mL, decrease in TgAb, and negative imaging.
- Biochemical incomplete response (BIR): suppressed Tg > 1 ng/mL or stimulated Tg > 10 ng/mL, increase in TgAb levels, and negative imaging.
- Structural incomplete response (SIR): Structural or functional evidence of disease with any Tg and TgAb levels. Besides, persistent SIR is defined as the reappearance of SIR within one year after ^131^I treatment, while emerging SIR is defined as the reappearance of disease one year after ^131^I treatment [16].

The responses to therapy were also defined as the acceptable response (AR), including patients with ER or IR, and the not acceptable response (NAR), including patients with BIR or SIR [28].

### 2.6. Statistical Analysis

Continuous data are expressed as mean ± standard deviation with range and median, and categorical data are shown as absolute number and percentage. Independent two-sample *t*-test was used for the comparison of continuous variables while *χ*^2^ and Fisher's exact tests for categorical variables. Multivariate logistic regression analysis was performed to identify factors significantly predicting responses to therapy. *p* < 0.05 was considered statistically significant. Statistical analyses were performed using IBM SPSS 21.0 (IBM Corp., Armonk, NY, USA) and Prism 7.0 (GraphPad Software, San Diego, CA, USA).

## 3. Results

### 3.1. Clinicopathological Characteristics of All Patients

The clinicopathological characteristics of 48 enrolled patients are listed in Table 1. The study population comprised 44 (91.67%) female and 4 (8.33%) male patients. The mean age at diagnosis was 16.3 ± 2.2 years (range, 9–18 years). The percentage of patients with tumor size >4 cm, multifocality, extra-thyroidal extension, and lymph node metastases were 16.67%, 45.83%, 35,42%, and 91.67%, respectively. Lung metastases were observed in three (6.25%) patients. Most of the patients were in the intermediate (*n* = 17, 35.42%) and high (*n* = 24, 50.00%) risk groups of the American Thyroid Association guidelines for children with DTC [2]. The mean cumulative dose of ^131^I was 135.63 ± 155.42 mCi (median, 100 mCi; range, 30–1150 mCi), and 40 (83.33%) patients accepted only one course of ^131^I treatment. TgAb levels decreasing ≥50% and TgAbs clearance were observed in 20 (58.33%) and 23 (45.83%) patients at the end of follow-up, respectively. The median follow-up period was 58 months (range, 24–143 months). There were no significant differences between the P-Tg and N-Tg groups for the baseline clinicopathological characteristics that we evaluated (Table 1), except that the stimulated Tg level was significantly higher in the P-Tg group than in the N-Tg group (*p*=.006).

### 3.2. Clinicopathologic Factors Associated with Responses to Therapy

At the end of follow-up, 28 (58.33%) patients had AR and 20 patients (41.37%) had NAR (Table 2). Responses to therapy at the end of follow-up were ER in 19 patients (1 patient with TgAb decreasing <50% and the others with TgAb decreasing ≥50%), IR in 9 patients (7 with TgAb decreasing ≥50% and 2 with TgAb decreasing <50%), BIR in 4 patients (1 with TgAb decreasing <50% and 3 with TgAb increasing), and SIR in 16 patients (3 with TgAb decreasing ≥50%, 4 with TgAb decreasing <50%, and 9 with TgAb increasing). Three patients had persistent lung metastases. Among these three patients, persistently high TgAb levels (TgAb > 4000 IU/ml) were observed in two patients, and decreasing Tg and TgAb levels were observed in another patient.

Univariate analysis showed that the extra-thyroidal extension (*p* < 0.001), high initial risk stratification (*p* < 0.001), TgAb decreasing <50% or TgAb increasing (*p* < 0.001), and consistently TgAb-positive (*p* < 0.001) were significantly associated with NAR (Table 2). However, other clinicopathologic variables were not associated with NAR (*p* > 0.05). Initial risk stratification and TgAb levels decreasing <50% or TgAb levels increasing were significantly associated with NAR according to multivariate logistic regression analysis (Table 3).

### 3.3. Clinicopathologic Factors Associated with Responses to Therapy in the P-Tg Group

The clinicopathological characteristics of 17 patients in the P-Tg group are listed in Table 4. All patients with positive Tg were female. The mean age at diagnosis was 16.59 ± 2.03 years (range, 12–18 years) and 4 (23.53%) patients were ≤15 years. The percentage of patients with tumor size >4 cm, multifocality, extra-thyroidal extension, and lymph node metastases were 17.64%, 47.16%, 47.16%, and 94.12%, respectively. Lung metastases were observed in only two (11.76%) patients. Ten (58.83%) patients were in the high initial risk stratification. The cumulative dose of ^131^I received by most patients (*n* = 12, 70.59%) was ≤100 mCi, and 13 (76.47%) patients accepted only one course of ^131^I treatment.

At the end of follow-up, 10 patients had AR and 7 patients had NAR in the P-Tg group (Table 4). Univariate analysis showed that the extra-thyroidal extension (*p*=0.029), high initial risk stratification (*p*=0.017), TgAb decreasing <50% or TgAb increasing (*p*=0.036), and Tg decreasing <50% or Tg increasing (*p*=0.036) were significantly associated with NAR in the P-Tg group patients (Table 4).

The mean TgAb levels decreased from 1321.46 ± 1698.76 IU/ml 6 months after initial ^131^I treatment to 984.90 ± 1640.95 IU/ml at the end of follow-up. TgAb levels were increasing in four patients (all with consistent SIR), decreasing <50% in three patients (two with consistent SIR), and decreasing ≥50% in 10 patients (one with emerging SIR). The mean Tg levels decreased from 2.07 ± 4.94 ng/ml 6 months after initial ^131^I treatment to 1.12 ± 2.43 ng/ml at the end of follow-up. Tg levels were increasing in three patients (one with consistent SIR and two with emerging SIR), decreasing <50% in three patients (two with consistent SIR), and decreasing ≥50% in 11 patients (two with consistent SIR and increasing TgAb).

### 3.4. Clinicopathologic Factors Associated with Responses to Therapy in N-Tg Groups

The clinicopathological characteristics of 17 patients in the P-Tg group are listed in Table 5. All patients with positive Tg were female. The mean age at diagnosis was 16.13 ± 2.247 years (range, 9–18 years) and 9 (29.03%) patients were≤15 years. The percentage of patients with tumor size >4 cm, multifocality, extra-thyroidal extension, and lymph node metastases were 16.13%, 45.16%, 29.03%, and 90.32%, respectively. Lung metastases were observed in only one (3.23%) patient. Twelve (38.71%) patients were in the high initial risk stratification. The cumulative dose of ^131^I received by most patients (*n* = 25, 80.65%) was ≤100 mCi, and 27 (87.09%) patients accepted only one course of ^131^I treatment.

At the end of follow-up, 18 (58.06%) patients had AR and 13 patients (41.94%) had NAR in the N-Tg group (Table 5). Univariate analysis showed that the extra-thyroidal extension (*p*=0.029), high initial risk stratification (*p*=0.003), TgAb decreasing <50% or TgAb increasing (*p* < 0.001), and consistently TgAb-positive (*p* < 0.001) were significantly associated with NAR in the N-Tg group.

The mean TgAb levels decreased from 741.73 ± 942.98 IU/ml 6 months after initial ^131^I treatment to 396.60 ± 811.32 IU/ml at the end of follow-up. TgAb levels were increasing in eight patients (three with emerging SIR, and two with consistent SIR), decreasing <50% in five patients (two with emerging SIR), and decreasing ≥50% in 18 patients (one with emerging SIR and one with consistent SIR). Tg-positive results were observed in two patients with TgAb increasing at the end of follow-up (one with emerging SIR and one with consistent SIR) and one patient with TgAb decreasing ≥50% without SIR.

### 3.5. Clinicopathologic Factors Associated with TgAb Trend

At the end of follow-up, the TgAb level decreasing ≥50% was observed in 29 (60.42%) patients while the TgAb level decreasing <50% or increased in 19 (39.58%) patients (Table 6). Univariate analysis showed that the extra-thyroidal extension (*p* < 0.001) and high initial risk stratification (*p* < 0.001) were significantly associated with the TgAb decreasing <50% or increasing. Although there was no significance, patients with TgAb decreasing <50% or increasing had higher N stage (*p*=0.062), higher M stage (*p*=0.056), and higher sTgAb (*p*=0.028).

### 3.6. Clearance Patterns of TgAbs

**Figure 1:**
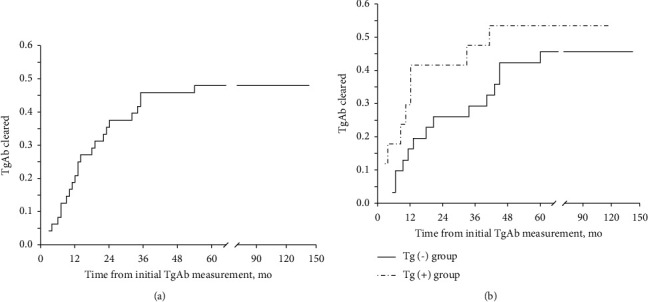
(a) Clearance of TgAb after initial treatment in pediatric patients with PTC. (b) Clearance of TgAb after initial treatment in pediatric patients with PTC in the P-Tg and N-Tg groups.

During follow-up, 23 (44%) patients became TgAb negative, with a mean time to clearance of 17.74 ± 2.715 months and a median time to clearance of 13 months (range: 3–54 months) (Figure 1(a)). Each of these 23 patients became TgAb negative in less than 3 years, except 1 patient who became TgAb negative after 54 months.

Among the 17 patients in the P-Tg group, 9 (52.9%) patients became TgAb negative, with a median time to clearance of 13 months (range, 3–54) (Figure 1(b)). Each of these 9 patients became TgAb negative in less than 3 years, 7/9 (77.8%) patients in less than 2 years, and 4/9 (44.44%) patients in less than 1 year. Among the 31 patients in the N-Tg group, 14 patients became TgAb negative, with a mean time to clearance of 20.36 ± 13.43 months (Figure 1(b)). Each of these 14 patients became TgAb negative in less than 3 years, except 1 patient who became TgAb negative after 54 months, 11/14 (78.57%) in less than 2 years, and 5/14 (35.71%) in less than one year. There was no significance in the time to TgAb clearance between the P-Tg and N-Tg groups.

## 4. Discussion

The focus of this study was to determine whether changes in TgAb levels over time could be used as a predictive indicator for responses to therapy in pediatric PTC patients. We studied the clinical outcomes of 48 TgAb-positive pediatric PTC patients. Our data show that changes in TgAb levels over time might be a good predictive indicator for responses to therapy in TgAb-positive pediatric PTC patients. We also show that changes in Tg levels could also be used as a predictive indicator in pediatric PTC patients who test positive for both Tg and TgAbs.

Complete elimination of follicular cells by total thyroidectomy and ^131^I treatment should lead to a progressive decrease in TgAbs due to the cessation of Tg secretion [13]. Therefore, persistent or increased TgAb levels after initial treatment may indicate the presence of abnormal Tg-secreting tissues, such as thyroid cancer cells. More specifically, the presence of TgAbs reflects persistent or recurrent DTC because all normal follicular cells have already been theoretically destroyed. Therefore, TgAb levels could be used as a surrogate tumor marker. When using TgAb as a surrogate tumor marker, the trend is more important than the absolute level [23, 29]. Many studies have reported that the de novo appearance or persistence of TgAbs or an increase in TgAb levels after initial treatment are significant risk factors for persistent or recurrent DTC. Responses to therapy, as measured by biochemical testing and structural or functional imaging, are closely associated with persistent or recurrent disease and have been recommended for surveillance of DTC[3]. Bueno et al. reported that changes in TgAb levels over time are more informative than an absolute TgAb value at a single time point in the prediction of SIR in patients with DTC after ^131^I treatment [17]. Although many studies have demonstrated the prognostic value of TgAbs in adult DTC patients, minimal data exist in pediatric PTC patients. To date, only Wassner et al. have reported the prevalence, clearance rates, and clinical significance of TgAb levels in pediatric thyroid cancer patients [19]. However, it is unclear whether the presence of TgAbs is an independent risk factor for persistent or recurrent DTC. Similar to previous studies in adult DTC patients [10, 13, 18, 25, 26, 30], our study shows that changes in TgAb levels could be a prognostic factor to predict responses to therapy of pediatric PTC patients. However, it should be noted that SIR was not observed in 25% of patients with persistently elevated TgAb levels in our study. One possible explanation for this may be the persistence of small amounts of normal or recurrent cancer tissue that escape detection by currently available diagnostic methods [23, 31]. Another possible explanation could be that the follow-up period may have been too short in some cases to demonstrate the complete disappearance of the antigenic stimulus or to detect recurrences. We also investigate the clinicopathologic factors associated with the TgAb trend. Our study showed that pediatric patients with TgAb decreasing <50% or increasing usually have invasive clinicopathologic factors, such as the extra-thyroidal extension and high initial risk stratification, which indicates the TgAb trend was associated with invasive clinicopathologic features in pediatric PTC patients. Therefore, we can speculate that changes in TgAb levels could be a prognostic factor in pediatric PTC patients from another perspective.

Previous studies on the clinical significance of TgAbs in PTC have generally focused on Tg-negative PTC patients, who have no theoretically possible Tg source. Although the existence of TgAb may interfere with Tg determinations, there are still some patients who had both positive Tg and TgAb. Whether changes in Tg levels could also be used as a prognostic factor in these patients remains unclear. In this study, we compared the clinicopathological characteristics of TgAb-positive pediatric PTC patients who were positive or negative for Tg. Our data show that TgAb-positive patients who were positive or negative for Tg had the same clinicopathological characteristics, except for the presence of stimulated Tg. In our study, Tg levels decreasing ≥50% and TgAb levels decreasing ≥50% are both significantly associated with AR in pediatric patients positive for both Tg and TgAbs. This indicates that changes in the levels of both Tg and TgAbs could predict the responses to therapy in TgAb-positive pediatric PTC patients positive for Tg. We also found that, of the TgAb-positive patients (*n* = 31) who were negative for Tg, three patients became Tg positive during follow-up, and among these patients, two had SIR at the end of follow-up. Therefore, although TgAbs may interfere with Tg determination, changes in Tg levels could also be used as a prognostic factor in TgAb-positive pediatric PTC patients. TgAb interferes with Tg immunometric assay (IMA) measurements, causing falsely low/undetectable Tg values. This problem would suggest that laboratories should adopt a dual strategy for serum Tg testing, which involved IMA, radioimmunoassay, and liquid chromatography-tandem mass spectrometry[7, 32]. However, this approach is impractical for routine clinical laboratories in our hospital. The patients in our study were divided into positive Tg group and negative Tg group according to suppressed Tg level measured by high-sensitivity electrochemiluminescence immunoassay method without using the dual strategy, which could have a slight influence on the current result. Therefore, further studies including a larger sample size are needed to determine whether surveillance of Tg and TgAb levels over time measured by a dual strategy could increase the prognostic value of biochemical measurements in pediatric PTC patients.

The prevalence of TgAbs in pediatric patients with thyroid cancer is higher than in adults [6, 19]. Wassner et al. reported that 41% of pediatric patients with thyroid cancer were TgAb positive, and TgAb positivity was observed in only 30.9% of pediatric DTC patients in our cohort [19]. TgAbs may reappear or increase in the 6 months after ^131^I treatment in some cases, owing to the release of Tg antigens secondary to radiolytic damage of thyroid tissue [12]. Therefore, the timing of TgAb measurements could influence the prevalence of positive TgAb values in patients after ^131^I treatment. To exclude the effect of transiently elevated TgAb levels after ^131^I treatment, the definition of positive TgAb levels in our study was based on samples taken 6 months after ^131^I treatment, which might have contributed to the lower prevalence of positive TgAb observed in our study compared with the previous study. Furthermore, our higher cutoff value for TgAb detection also contributed to lower prevalence compared with previous pediatric studies [19].

TgAbs decrease and eventually disappear after initial treatment in about 30% of patients because the source of Tg has been completely removed, although the clearance may take up to 3 years [33]. Wassner et al. reported that TgAb clearance occurred in 44% of pediatric DTC patients over a median follow-up time of 3.8 years [19]. In their study, the median time to TgAb clearance was 10.7 months, and 10 out of 11 patients had TgAb clearance within 2 years. In our study, the median time for TgAb clearance was 18 months, which is higher than in the previous study by Wassner et al. [19]. The prevalence of lymph node metastasis was higher than in patients reported in previous studies [19, 34], which may have caused the longer clearance times observed in our study.

There were some limitations to this study. First, the study is retrospective in design, and the long follow-up time may have caused deviations in data selection. Second, a large proportion of our patients were >15 years old, so the clinicopathological characteristics and management procedures could be more similar to adult patients. Third, because our definition of positive TgAb levels was based on measurements performed 6 months after ^131^I treatment, the clinical significance of TgAb levels in TgAb-positive patients during the 6-month time period after ^131^I treatment is not clear and needs further study. Fourth, in this study, we did not include patients who have the *de novo* detection of TgAb after ^131^I treatment, and further studies are needed to determine whether the TgAb trend could also be used as a prognostic factor in these pediatric PTC patients.

## 5. Conclusion

In conclusion, this is the first study to report the prognostic value of TgAbs in pediatric PTC patients. We found that changes in TgAb levels over time could be used as a predictor of responses to therapy in TgAb-positive pediatric PTC patients. We also found that changes in Tg levels over time are associated with responses to therapy in pediatric PTC patients. Therefore, we recommend that a more aggressive imaging test should be performed for pediatric PTC patients with elevated or persistent TgAb levels. Although our data suggest that changes in TgAb levels could be used as a surrogate tumor marker in TgAb-positive pediatric PTC patients, larger prospective studies are required to account for additional potential confounding variables.

## Figures and Tables

**Table 1:** Clinicopathological characteristics of all patients.

	Total	Tg negative	Tg positive	*p*

Age at diagnosis of DTC				
≤15	13 (27.08%)	9 (29.03%)	4 (23.53%)	0.944
>15	35 (72.92%)	22 (70.97%)	13 (76.47%)	
Sex				
Female	44 (91.67%)	27 (87.10%)	17 (100%)	0.317
Male	4 (8.33%)	4 (12.90%)	0 (0%)	
Number of thyroid surgeries				
1	40 (83.33%)	27 (87.10%)	13 (76.47%)	0.589
>1	8 (16.67%)	4 (12.90%)	4 (23.53%)	
Tumor diameter (cm)				
≤2	24 (50.0%)	17 (54.84%)	7 (41.18%)	0.634
>2, ≤4	16 (33.33%)	9 (29.03%)	7 (41.18%)	
>4	8 (16.67%)	5 (16.13%)	3 (17.64%)	
Multiplicity				
Single	26 (54.17%)	17 (54.84%)	9 (52.94%)	0.900
Multiple	22 (45.83%)	14 (45.16%)	8 (47.06%)	
Extra-thyroidal extension				
No	31 (64.58%)	22 (71.97%)	9 (52.94%)	0.212
Minimal/gross	17 (35.42%)	9 (29.03%)	8 (47.06%)	
N stage				
N0 or Nx	4 (8.33%)	3 (9.68%)	1 (5.88%)	0.902
N1a	11 (22.92%)	7 (22.58%)	4 (23.53%)	
N1b	33 (68.75%)	21 (66.74%)	12 (70.59%)	
Distant metastases				
No	45 (93.75%)	30 (96.77%)	15 (88.24%)	0.585
Yes	3 (6.25%)	1 (3.21%)	2 (11.76%)	
Initial risk stratification				
Low	7 (14.58%)	5 (16.13%)	2 (11.74%)	0.406
Intermediate	17 (35.42%)	14 (45.16%)	5 (29.41%)	
High	24 (50.00%)	12 (38.71%)	10 (58.82%)	
With LT				
No	25 (52.08%)	54.84 (%)	8 (47.06%)	0.606
Yes	23 (47.92%)	45.16 (%)	9 (52.94%)	
Number of courses for ^131^I therapy				
1	40 (83.33%)	27 (87.10%)	13 (76.47%)	0.589
>1	8 (16.67%)	4 (12.90%)	4 (23.53%)	
Cumulative dose of ^131^I activities (mCi)				
≤100	37 (77.08%)	25 (70.59%)	12 (80.65%)	0.664
>100	11 (22.92%)	6 (29.41%)	5 (19.35%)	
sTg (ng/ml)				
<1	32 (66.67%)	25 (80.65%)	7 (41.18%)	0.006 ^*∗*^
≥1	16 (33.33%)	6 (19.35%)	10 (58.82%)	
sTgAb (IU/ml)				
<500	27 (56.25%)	16 (51.61%)	11 (64.71%)	0.382
≥500	21 (43.75%)	15 (48.39%)	6 (35.29%)	
TgAb trend				
Decrease ≥50%	28 (58.33%)	18 (50.06%)	11 (64.71%)	0.653
Decrease <50% or increase	20 (41.67%)	13 (41.94%)	6 (35.29%)	
TgAb clearance				
Yes	23 (45.83%)	14 (45.16%)	9 (52.94%)	0.606
No	25 (54.17%)	17 (54.84%)	8 (47.06%)	
Follow-up time	63.81 ± 31.25	57.81 ± 29.11	74.76 ± 32.90	0.086

sTg, stimulated Tg; sTgAb, stimulated TgAb; N, lymph node; LT, lymphocytic thyroiditis.  ^*∗*^*p* < 0.05.

**Table 2:** Univariate analysis clinicopathologic factors associated with responses to therapy.

	No. of patients	Acceptable	Unacceptable	*p*

Age at diagnosis of DTC				
≤15	13 (27.08%)	7 (25.00%)	6 (30.00%)	0.701
>15	35 (73.93%)	21 (75.00%)	14 (70.00%)	
Sex				
Female	44 (91.67%)	25 (89.29%)	19 (95.00%)	0.860
Male	4 (8.3%)	3 (10.71%)	1 (5.00%)	
Number of thyroid surgeries				
1	40 (83.33%)	24 (85.71%)	16 (80.00%)	0.896
>1	8 (16.67%)	4 (14.29%)	4 (20.00%)	
Tumor diameter (cm)				
≤2	24 (50.0%)	15 (53.57%)	9 (45.00%)	0.807
>2, ≤4	16 (33.33%)	9 (32.14%)	7 (35.00%)	
≥4	8 (16.67%)	4 (14.27%)	4 (10.00%)	
Multiplicity				
Single	26 (54.17%)	16 (57.14%)	10 (50.00%)	0.624
Multiple	22 (45.83%)	12 (42.86%)	10 (50.00%)	
Extra-thyroidal extension				
No	31 (64.58%)	24 (85.71%)	7 (35.00%)	＜0.001 ^*∗*^
Minimal/gross	17 (35.42%)	4 (14.28%)	13 (65.00%)	
N stage				
N0 or Nx	4 (8.33%)	2 (7.14%)	2 (10.00%)	0.536
N1a	11 (22.92%)	8 (28.57%)	3 (15.00%)	
N1b	33 (68.75%)	18 (64.29%)	15 (75.00%)	
Initial risk stratification				
Low/intermediate	26 (54.17%)	22 (78.57%)	4 (20.00%)	＜0.001 ^*∗*^
High	22 (45.83%)	32 (21.43%)	16 (80.00%)	
With LT				
No	25 (52.08%)	13 (46.43%)	12 (60.00%)	0.353
Yes	23 (47.92%)	15 (53.57%)	8 (40.00%)	
Number of courses for ^131^I therapy				
1	40 (83.33%)	24 (85.71%)	16 (80.00%)	0.896
>1	8 (16.67%)	4 (14.29%)	4 (20.00%)	
Cumulative dose of ^131^I activities (mCi)				
≤100	40 (83.33%)	24 (85.71%)	13 (65.00%)	0.182
>100	8 (16.67%)	4 (14.29%)	7 (14.29%)	
sTg (ng/ml)				
<1	32 (66.67%)	19 (67.86%)	13 (65.00%)	0.836
≥1	18 (33.33%)	9 (32.14%)	7 (35.00%)	
sTgAb (IU/ml)				
<500	27 (56.25%)	19 (67.86%)	8 (40.00%)	0.055
≥500	21 (43.75%)	9 (32.14%)	12 (60.00%)	
Suppressed Tg at first follow-up after RAI therapy				
Tg negative	31 (64.58%)	18 (64.29%)	13 (65.00%)	0.959
Tg positive	17 (35.41%)	10 (35.71%)	7 (35.00%)	
TgAb trend				
Decrease ≥50%	28 (58.33%)	25 (89.28%)	4 (20.00%)	＜0.001 ^*∗*^
Decrease <50% or increase	20 (41.67%)	3 (10.71%)	16 (80.00%)	
TgAb tends to be negative				
Yes	23 (47.92%)	20 (28.57%)	3 (15.00%)	＜0.001 ^*∗*^
No	25 (52.08%)	8 (71.43%)	17 (85.00%)	

sTg, stimulated Tg; sTgAb, stimulated TgAb; N, lymph node; LT, lymphocytic thyroiditis.  ^*∗*^ *p* < 0.05.

**Table 3:** Multivariate logistic regression analysis for clinicopathologic factors associated with responses to therapy.

	OR	95% CI	*p*

Extra-thyroidal extension (no/minimal or gross)			0.294
Initial risk stratification (low/intermediate or high)	6.578	1.188–36.421	0.031 ^*∗*^
sTgAb (<500/≥500 IU/ML)			0.833
TgAb trend (decrease ≥50%/decrease <50% or increase)	18.847	3.345–106.188	0.001 ^*∗*^
TgAb tends to be negative (yes/no)			0.142

sTg, stimulated Tg; sTgAb, stimulated TgAb.  ^*∗*^*p* < 0.05.

**Table 4:** Univariate analysis for clinicopathologic factors associated with responses to therapy in P-Tg groups.

	No. of patients	Acceptable	Unacceptable	*p*

Age at diagnosis of DTC				
≤15	4 (23.53%)	(30.00%)	1 (14.29%)	0.864
>15	13 (76.47%)	(70.00%)	6 (85.71%)	
Sex				
Female	17 (100.0%)	10 (100.00%)	7 (100.00%)	
Number of thyroid surgeries				
1	13 (76.47%)	9 (90.0%)	4 (57.14%)	0.322
>1	4 (23.53%)	1 (10.0%)	3 (42.86%)	
Tumor diameter (cm)				
≤2	7 (41.18%)	5 (50.0%)	2 (28.57%)	0.529
>2, ≤4	7 (41.18%)	4 (40.0%)	3 (42.86%)	
>4	3 (17.64%)	1 (10.0%)	2 (28.57%)	
Multiplicity				
Single	9 (52.94%)	6 (60.0%)	3 (42.86%)	0.839
Multiple	8 (47.16%)	4 (40.0%)	4 (57.14%)	
Extra-thyroidal extension				
No	9 (52.94%)	8 (80.0%)	1 (14.29%)	0.029 ^*∗*^
Minimal/gross	8 (47.16%)	2 (20.0%)	6 (85.71%)	
N stage				
N0 or Nx	1 (5.88%)	0 (0%)	1 (14.29%)	0.100
N1a	4 (23.53%)	4 (40.0%)	0 (0%)	
N1b	12 (70.59%)	6 (60.0%)	6 (85.71%)	
Initial risk stratification				
Low/intermediate	7 (41.18%)	7 (70.0%)	0 (0%)	0.017 ^*∗*^
High	10 (58.82%)	3 (30.0%)	7 (100.0%)	
With LT				
No	9 (52.94%)	5 (50.0%)	3 (42.86%)	1.000
Yes	8 (47.16%)	5 (50.0%)	4 (57.14%)	
Number of courses for ^131^I therapy				
1	13 (76.47%)	8 (80.0%)	5 (71.43%)	1.000
>1	4 (23.53%)	2 (20.0%)	2 (28.57%)	
Cumulative dose of ^131^I activities (mCi)				
≤100	12 (70.59%)	8 (80.0%)	4 (57.14%)	0.633
>100	5 (29.41%)	2 (20.0%)	3 (42.86%)	
sTg (ng/ml)				
<1	7 (41.18%)	4 (40.0%)	3 (42.86%)	1.000
≥1	10 (58.82%)	6 (60.0%)	4 (57.14%)	
sTgAb (IU/ml)				
<500	11 (64.71%)	8 (80.0%)	3 (42.86%)	0.288
≥500	6 (35.29%)	2 (20.0%)	4 (57.14%)	
TgAb trend				
Decrease >50%	11 (64.71%)	9 (90.0%)	2 (28.57%)	0.036 ^*∗*^
Decrease <50% or increase	6 (35.29%)	1 (10.0%)	5 (71.43%)	
TgAb tends to be negative				
Yes	8 (47.06%)	3 (30.0%)	5 (71.43%)	0.234
No	9 (52.94%)	7 (70.0%)	2 (28.57%)	
Tg trend				
Decrease ≥50%	11 (64.71%)	9 (90.0%)	2 (28.57%)	0.036 ^*∗*^
Decrease <50% or increase	6 (35.29%)	1 (10.0%)	5 (71.43%)	
Tg tends to be negative				
Yes	7 (41.18%)	2 (20.0%)	5 (71.43%)	0.105
No	10 (58.82%)	8 (80.0%)	2 (28.57%)	

sTg, stimulated Tg; sTgAb, stimulated TgAb; N, lymph node; LT, lymphocytic thyroiditis.  ^*∗*^*p* < 0.05.

**Table 5:** Univariate analysis for clinicopathologic factors associated with responses to therapy in N-Tg groups.

	No. of patients	Acceptable	Unacceptable	*p*

Age at diagnosis of DTC				
≤15	9 (29.03%)	4 (22.22%)	5 (38.46%)	0.433
>15	22 (70.97%)	14 (77.78%)	8 61.54%)	
Sex				
Female	27 (100.0%)	15 (83.33%)	12 (92.31%)	0.847
Male	4 (100.0%)	3 (16.67%)	1 (7.69%)	
Number of thyroid surgeries				
1	27 (87.10%)	15 (83.33%)	12 (92.30%)	0.621
>1	4 (12.90%)	3 (16.67%)	1 (7.69%)	
Tumor diameter (cm)				
≤2	17 (54.84%)	10 (55.56%)	7 (53.85%)	0.983
>2, ≤4	9 (29.03%)	5 (27.78%)	4 (30.77%)	
>4	5 (16.13%)	3 (16.67%)	2 (15.38%)	
Multiplicity				
Single	17 (54.84%)	10 (55.56%)	3 (53.85%)	0.925
Multiple	14 (45.16%)	8 (44.44%)	4 (46.15%)	
Extra-thyroidal extension				
No	22 (71.97%)	16 (88.89%)	6 (46.15%)	0.029 ^*∗*^
Minimal/gross	9 (29.03%)	2 (11.11%)	7 (53.85%)	
N stage				
N0 or Nx	3 (9.68%)	2 (11.11%)	1 (7.69%)	0.171
N1a	7 (22.58%)	4 (22.22%)	3 (23.08%)	
N1b	21 (67.74%)	12 (66.67%)	9 (69.23%)	
Initial risk stratification				
Low/intermediate	19 (63.29%)	15 (83.33%)	4 (30.78%)	0.003 ^*∗*^
High	12 (38.71%)	3 (16.67%)	9 (69.23%)	
With LT				
No	17 (54.84%)	8 (44.44%)	9 (69.23%)	0.171
Yes	14 (45.16%)	10 (55.56%)	4 (30.78%)	
Number of courses for ^131^I therapy				
1	27 (87.09%)	16 (88.89%)	11 (84.62%)	1.000
>1	4 (12.90%)	2 (11.11%)	2 (15.38%)	
Cumulative dose of ^131^I activities (mCi)				
≤100	25 (80.65%)	16 (88.89%)	9 (69.23%)	0.365
>100	6 (19.35%)	2 (11.11%)	4 (30.78%)	
sTg (ng/ml)				
<1	25 (80.65%)	15 (83.33%)	10 (76.92%)	1.000
≥1	6 (19.35%)	3 (16.67%)	3 (23.08%)	
sTgAb (IU/ml)				
<500	16 (51.61%)	11 (61.11%)	5 (38.46%)	0.288
≥500	15 (48.39%)	7 (38.89%)	8 (61.54%)	
TgAb trend				
Decrease ≥50%	18 (64.71%)	16 (88.89%)	2 (15.38%)	<0.001 ^*∗*^
Decrease <50% or increase	13 (35.29%)	2 (11.11%)	11 (84.62%)	
TgAb tends to be negative				
Yes	17 (54.84%)	5 (27.78%)	5 (92.31%)	<0.001 ^*∗*^
No	14 (45.16%)	13 (72.22%)	2 (7.69%)	

sTg, stimulated Tg; sTgAb, stimulated TgAb; N, lymph node; LT, lymphocytic thyroiditis.  ^*∗*^*p* < 0.05.

**Table 6:** Clinicopathologic factors associated with the TgAb trend.

	No. of patients	Decrease ≥50%	Decrease <50% or increase	*p*

Age at diagnosis of DTC				
≤15	13 (27.08%)	7 (24.14%)	6 (31.58%)	0.571
>15	35 (73.93%)	22 (75.86%)	13 (68.42%)	
Sex				
Female	44 (91.67%)	26 (89.66%)	18 (94.74%)	1.000
Male	4 (8.3%)	3 (10.34%)	1 (5.26%)	
Tumor diameter (cm)				
≤2	24 (50.0%)	17 (58.62%)	7 (36.84%)	0.227
>2, ≤4	16 (33.33%)	9 (31.03%)	7 (36.84%)	
>4	8 (16.67%)	3 (10.34%)	5 (26.32%)	
Multiplicity				
Single	26 (54.17%)	17 (58.62%)	9 (47.37%)	0.444
Multiple	22 (45.83%)	12 (41.38%)	10 (52.63%)	
Extra-thyroidal extension				
No	31(64.58%)	24 (82.76%)	7 (36.84%)	<0.001 ^*∗*^
Minimal/gross	17(35.42%)	5 (17.24%)	12 (63.16%)	
N stage				
N0 or Nx	4 (8.33%)	2 (6.90%)	2 (10.53%)	0.062
N1a	11 (22.92%)	10 (34.48%)	1 (5.26%)	
N1b	33 (68.75%)	17 (56.62%)	16 (84.21%)	
Distant metastases				
No	45 (93.75%)	29 (100.0%)	16 (84.21%)	0.056
Yes	3 (6.25%)	0 (0%)	2 (15.79%)	
Initial risk stratification				
Low	7 (14.58%)	5 (17.24%)	2 (10.53%)	<0.001 ^*∗*^
Intermediate	19 (39.58%)	17 (58.62%)	2 (10.53%)	
High	22 (45.83%)	7 (24.14%)	15 (78.95%)	
With LT				
No	25 (52.08%)	14 (48.28%)	11 (57.89%)	0.514
Yes	23 (47.92%)	15 (51.72%)	8 (42.11%)	
Number of courses for ^131^I therapy				
1	40 (83.33%)	25 (86.21%)	15 (78.95%)	0.695
>1	8 (16.67%)	4 (13.79%)	4 (21.05%)	
Cumulative dose of ^131^I activities (mCi)				
≤100	37 (77.08%)	24 (82.76%)	13 (68.42%)	0.304
>100	11 (22.92%)	5 (17.24%)	6 (31.58%)	
Suppressed Tg at first follow-up after RAI therapy				
Tg negative	31 (64.58%)	18 (62.07%)	13 (68.42%)	0.653
Tg positive	17 (35.42%)	11 (37.93%)	6 (31.58%)	
sTg (ng/ml)				
<1	32 (66.67%)	20 (68.97%)	12 (63.16%)	0.676
≥1	16 (33.33%)	9 (31.03%)	7 (36.84%)	
sTgAb (IU/ml)				
<500	27 (56.25%)	20 (68.97%)	7 (36.84%)	0.028
≥500	21 (43.75%)	9 (31.03%)	12 (63.16%)	

sTg, stimulated Tg; sTgAb, stimulated TgAb; N, lymph node; LT, lymphocytic thyroiditis.  ^*∗*^*p* < 0.05.

## Data Availability

Some or all datasets generated during and/or analyzed during the current study are not publicly available but are available from the corresponding author on reasonable request.
